# Enhanced Silver Nanowire Composite Window Electrode Protected by Large Size Graphene Oxide Sheets for Perovskite Solar Cells

**DOI:** 10.3390/nano9020193

**Published:** 2019-02-02

**Authors:** Hongye Chen, Min Li, Xiaoyan Wen, Yingping Yang, Daping He, Wallace C. H. Choy, Haifei Lu

**Affiliations:** 1 School of Science, Wuhan University of Technology, Wuhan 430070, China; hongyechen@whut.edu.cn (H.C.); minli@whut.edu.cn (M.L.); wenxy@whut.edu.cn (X.W.); ypyang@whut.edu.cn (Y.Y.); hedaping@whut.edu.cn (D.H.); 2 Department of Electrical and Electronic Engineering, The University of Hong Kong, Pok Fu Lam Road, Hong Kong SAR, China; chchoy@eee.hku.hk

**Keywords:** Ag nanowires, graphene oxide, perovskite solar cell, transparent bottom electrode

## Abstract

Despite the outstanding features of high transmittance and low sheet resistance from silver nanowire (Ag NW) based transparent electrodes, their applications in perovskite solar cells (PVSCs) as window electrodes encounter significant obstacles due to the stability issue brought by the corrosion of halogen species from perovskite layer. In this study, we used large size graphene oxide (LGO) sheets as the protective barrier for bottom Ag NW nano-network. Contributed by the LGO with average size of 60 μm, less GO sheet was necessary for forming the fully covered protective barrier with fewer cracks, which consequently improved the optical transparency and anticorrosive ability of the composite electrode compared to the one from relatively small size GO. Our experiments demonstrated the composite electrode of Ag NW/LGO. The glass substrate exhibited transmittance of 83.8% and 81.8% at 550 nm before and after partial reduction, which maintained 98.4% and 95.1% average transmittance (AVT) of the pristine Ag NW electrode. Meanwhile, we utilized the steady hot airflow to assist the fast solvent evaporation and the uniform GO film formation on Ag NW electrode. Before the application of composite electrode in organic-inorganic hybrid perovskite solar cells, the operational stability of composite electrodes from different sizes of GO with perovskite film fabricated on top were characterized under continuing external bias and light irradiation. Experimental results indicate that the Ag NW electrode protected by LGO could maintain original resistance for more than 45 h. Finally, the PVSC fabricated on Ag NW/LGO based composite electrode yielded a power conversion efficiency (PCE) of 9.62%, i.e., nearly 85% of that of the reference device fabricated on the commercial indium-tin oxide (ITO) glass. Our proposed low temperature and solution processed bottom electrode with improved optical transparency and operational stability can serve as the very beginning layer of optoelectronic devices, to promote the development of low cost and large area fabrication perovskite solar cells.

## 1. Introduction

Organic-inorganic hybrid perovskite has been demonstrated as a promising candidate of light absorber because of its excellent properties for photovoltaic devices. The power conversion efficiency (PCE) has achieved over 23% since it was first introduced in 2009 with PCE of 3.8% [1,2,3,4,5,6,7]. With the increase in efficiency and potential commercial value, researchers have paid more attention to controlling the cost of preparation, by avoiding the use of high energy consuming instruments and adopting low temperature and solution processed methods for each layer. For example, the low temperature and solution processed interfacial layers, including electron [8,9,10,11,12,13,14] and hole [15,16,17,18,19] transporting layers, have been intensively studied to improve the efficiency of PVSCs. Carbon based materials, such as carbon paste [20,21], carbon nanotubes [22,23,24,25] and graphene [26], have also been used as top electrodes in PVSCs to replace conventional top metal electrodes formed by thermal evaporation, to reduce the cost of device fabrication and improve the stability of device simultaneously. Besides, metal nanowire electrode through spray-coating or spin-coating method is another favored candidate of top electrode for semi-transparent PVSCs [27,28].

For the bottom electrode of PVSCs, the most commonly used transparent conductive electrodes at present are indium-tin oxide (ITO) and fluorine-doped tin oxide (FTO) films fabricated by evaporation or sputtering in high vacuum environment, which however have the following problems. First, due to the large amount of electrical energy consumption and machine loss during the preparation process, the fabrication cost of ITO and FTO is relatively high. Second, the indium element required for ITO is a rare element. The rising of its price could be predicted in the long-term. Third, the metal oxide electrode has natural brittleness. The optoelectronic device based on this type of electrode will be greatly limited in flexibility and folding characteristics. As a result, several candidate materials have been investigated as bottom electrode to replace ITO or FTO, such as carbon nanotubes [29], graphene [30,31], conductive polymers [32] and metal nanowires [33,34,35,36]. Among these materials, silver nanowire (Ag NW) network is regarded as the most promising candidate because of the low sheet resistance and high transmittance. Moreover, silver nanowires have excellent flexibility, making them suitable for flexible optoelectronic device. However, the application of silver nanowire as bottom electrode to perovskite solar cells has encountered various obstacles, especially the chemical stability upon the perovskite materials because the halide species released from the perovskite layer will easily penetrate through the conventional interfacial layer, corrode the bottom silver nanowires, and thus lead to degradation of the electrical conduction [37,38,39].

To address the chemical stability issue, various attempts have been made to protect the bottom silver nanowire electrode. For instance, Han et al. [38] proposed a dense fluorine-doped ZnO (FZO) layer deposited on the surface of Ag NWs by pulsed laser deposition (PLD) to enhance the corrosion resistance of the electrode and ensure the conductivity of the electrode, achieving a PCE of 3.29% from perovskite device. Kim et al. [39] demonstrated a sandwich protection structure of ITO/Ag NW/ITO by successive-spin-coating method and annealing at 250 °C to enhance the conductivity of the composite electrode, resulting in a PCE of 8.44% from PVSC. Based on the sandwich protection structure, Lee et al. [37] proposed using amorphous Al-doped zinc oxide (AZO) instead of ITO as a protective layer for the purpose of reducing the use of indium and achieved a PCE of 13.93% from PVSC. In addition, our previous research [40] reported an anti-corrosive film composed of self-assembled graphene oxide (GO) flakes on a silver nano-network under ambient conditions, which can effectively prevent halide corrosion against the Ag NWs, and finally revealed a PCE of 9.23% by optimizing the amount of graphene oxide and reducing agent.

We demonstrated the formation of fully covered film composed of large size graphene oxide sheets (LGO) as the protective barrier for bottom Ag NW nano-network through utilizing a time-saving strategy of steady hot airflow, which is beneficial for the film formation with good uniformity. We found that the anti-corrosive abilities of the composite Ag NW electrodes from large size GO sheets were significantly enhanced compared to those composited from small size GO sheets. They were experimentally demonstrated by characterizing the resistance of composite electrode under continuing external bias and light irradiation. Importantly, the composite electrode of Ag NW/LGO after partially reduction showed an excellent optical transmittance, reaching 81.8% at 550 nm and maintaining 95.1% average transmittance (AVT) of the pristine Ag NW electrode. Finally, the PVSC fabricated with Ag NW/LGO based composite electrode demonstrated a power conversion efficiency (PCE) of 9.62%, corresponding to nearly 85% of the efficiency from reference device on commercial ITO glass.

## 2. Materials and Methods 

### 2.1. Materials

Graphene oxide powder was purchased from Anhui Lianruan Education Technology Co., Ltd. (Hefei, China). ITO glasses were purchased from YINGKOU OPV TECH NEW ENERGY Co., Ltd. (Yingkou, China). Poly(3,4-ethylenedioxythiophene):poly(styrene sulfonate) PEDOT:PSS (Heraeus Clevios PVP AI 4083), Lead (II) iodide(PbI_2_, >99.99%) and 2,9-dimethyl-4,7-diphenyl-1,10-Phenanthroline (BCP, >99%) were purchased from Xi’an Polymer Light Technology Corp. (Xian, China). Methylammonium iodide (MAI, 99%) was purchased from Greatcell Solar PTY Ltd. (Queanbeyan, Australia). [6,6]-Phenyl-C61- butyric acid methyl ester (PCBM, >99.5%) was purchased from Luminescence Technology Corp. (Shanghai, China). Dimethyl sulfoxide (DMSO, anhydrous, ≥99.9%), chlorobenzene (anhydrous, 99.8%) and 2-Propanol (anhydrous, 99.5%) were purchased from Sigma-Aldrich Co. LLC. (Shanghai, China). γ-Butyrolactone (GBL, anhydrous, 99.9%) was purchased from Shanghai Aladdin Biochemical Technology Co., Ltd. (Shanghai, China). Ethanol, acetone and sodium borohydride were purchased from Sinopharm Chemical Reagent Co., Ltd. (Shanghai, China). All chemicals were used without further purification.

### 2.2. Preparation of Large Size GO

Large size GO sheets were prepared using a centrifugal classification method according to previous report [41]. Briefly, appropriate amount of GO powder was taken for preparing an aqueous dispersion with a concentration of 2 mg/mL, which was stirred for 24 h. After centrifugation at 5000 rpm for 30 min, the bottom solution (about 30% in volume) was kept and diluted to 2 mg/mL for the next cycle of centrifugation. After repeating the above process seven times, the gel at bottom was collected and used for investigations in this study.

### 2.3. Preparation of Ag NW/RLGO Composite Electrodes

The glass substrates were cleaned ultrasonically with acetone, deionized water and ethanol for 15 min in sequence, and then dried with N_2_, followed by UV/ozone treatment for 15 min. The pristine Ag NW dispersion was diluted to 1.3 mg/mL before use. Highly conductive Ag NW transparent electrodes were fabricated by spin-coating the Ag NW dispersion at 2500 rpm for 30 s and annealing at 150 °C for 10 min in air. According to our previous research [40], 32 μmol NaBH_4_ were added to 2.5 mL LGO aqueous dispersion (0.25 mg/mL). Then, the dispersion was kept for 12 h for the partial reduction of LGO. Fifty microliters of partially-reduced LGO dispersion (RLGO) were dropped onto the Ag NW transparent electrode with area of 2.89 cm^2^, allowing the liquid to disperse onto the entire substrate uniformly. Finally, the substrate was dried under a steady hot airflow, which could greatly speed up the evaporation of the solution. The “steady hot air flow” was achieved by a commercial adjustable electric hair dryer (FH6618, FLYCO, Shanghai, China) through carefully controlling wind and power.

### 2.4. Preparation of Perovskite Solar Cells on Ag NW/RLGO Composite Electrodes

For the fabrication of perovskite solar cells, the transparent electrodes of Ag NW/RLGO and commercial ITO glass were used. The Ag NW/RLGO composite transparent electrodes were prepared as described above. The control ITO substrates were ultrasonically cleaned with detergent, deionized water, acetone, and ethanol for 15 min in sequence. To prepare hole transport layer, the as-received PEDOT:PSS solution was spin-coated on the transparent electrodes at a speed of 2000 rpm for 30 s and annealed on a hotplate at 125 °C for 10 min. The samples were then transferred to a glove-box after cooling to room temperature. The perovskite MAPbI_3_ films were fabricated on the substrates according to a previously reported method [42]. Generally, PbI_2_ and MAI with 1:1 ratio were mixed in the mixing solvent of GBL and DMSO (volume ratio = 7:3) to form the precursor with a concentration of 1.2 M. The mixture solution was stirred at 60 °C overnight before use. The perovskite films were fabricated by a successive two step spin-coating process of 1000 rpm for 10 s and 4000 rpm for 30 s. During the second step of spin-coating, 150 μL of toluene were quickly dropped on the substrate, which was then annealed on a hotplate at 100 °C for 10 min. The PCBM layer was deposited on the as-formed perovskite film by spin-coating its chlorobenzene solution (17 mg/mL) at 2000 rpm for 30 s, followed by annealing at 100 °C for 10 min. Subsequently, a thin BCP film, used as buffer layer, was deposited by spin-coating its saturated solution at 3000 rpm for 60 s. Finally, the device was completed by thermal evaporation of 150 nm thick Ag cathode under 3.0 × 10^−4^ Pa.

### 2.5. Characterizations

The surface morphology and cross section of the as-prepared samples were examined by field-emission scanning electron microscope (FE-SEM, Zeiss Ultra Plus, Oberkochen, German). GO sheets were measured by optical microscope (XJP-107JX, Pudan Optical Instrument Co., Ltd., Shanghai, China). The diffused transmission spectra of the transparent electrodes were obtained from a UV-vis spectrophotometer (UV2600, Shimadzu, Tokyo, Japan) with an integrating sphere. The sheet resistances of the electrodes were measured using a four-point probe system with a current source-meter (Keithley 2400, Tektronix, Beaverton, OR, USA). To characterize the resistance variation of composite electrode before and after device fabrication, two electrical contacts were formed on the two sides of the electrode using silver paste and a digital multimeter (DT9206, FLUKE Corporation, Elite, WA USA) was used for resistance measurement. The photocurrent density–voltage (J–V) characteristics of all devices were analyzed using an AM1.5G solar simulator (Oriel Sol3A, Newport Corporation, Irvine, CA, USA) and Keithley 2400 sourcemeter (Beaverton, OR, USA), scanning from −0.1 to 1.2 V at a scan rate of 0.1 V s^−1^. The IPCE (Newport Corporation, Irvine, CA, USA) system was employed to study the quantum efficiency of the solar cells.

## 3. Results and Discussion

**Figure 1:**
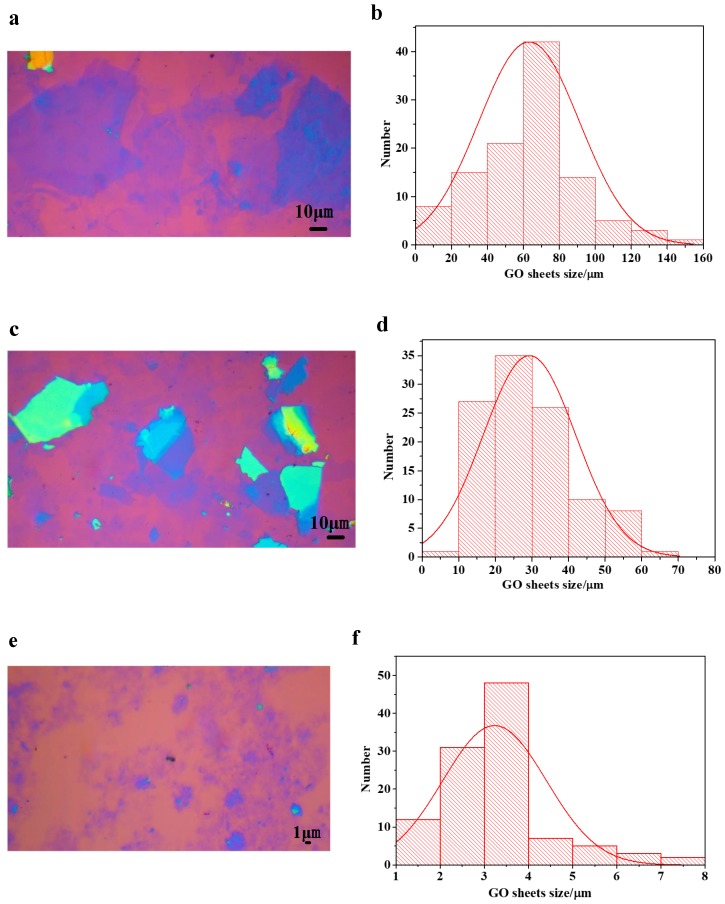
(**a**,**c**,**e**) The optical microscope images of 60 μm, 30 μm and 3 μm GO sheets, respectively;and (**b**,**d**,**f**) the size distributions of 60 μm, 30 μm and 3 μm GO sheets, respectively.

In our previous research [40], we demonstrated that Ag NWs underneath perovskite layer was severely corroded after the formation of perovskite film, which is the most critical issue that hinders their application in PVSCs as bottom electrode. To solve the chemical instability and improve the optical transparency, we used a fully-covered film composed of LGO sheets on silver nanowire electrode as an anti-corrosive barrier. As depicted in Figure 1, three different GO sheets with average sizes of 60 μm, 30 μm and 3 μm were successfully separated by a centrifugal classification method. The obtained GO sheets were diluted to an appropriate concentration, dropped on clean silicon wafer with a 300 nm SiO_2_ layer, and then characterized by optical microscope. In Figure 1, we can clearly observe that most GO sheets were thin and well classified, which was beneficial to the transmittance of Ag NW/GO composite electrodes and our later research. Figure 1b,d,f shows the size distributions of the three different GO sheets.

**Figure 2:**
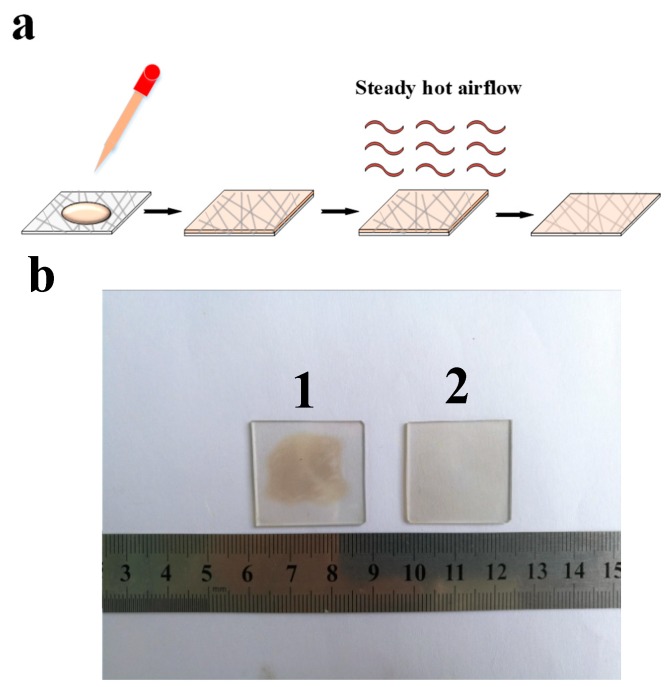
(**a**) Schematic representation of the Ag NW/LGO composite electrode fabrication process; and (**b**) photos of Ag NW/LGO composite electrodes fabricated from relatively high concentration of LGO solution (0.5 mg/mL) on the area of 6.25 cm^2^ using two different methods.

It is also worth mentioning our strategy of forming fully-covered GO film on Ag NW substrate. Our previous method of forming the GO film was obtained through self-assembly and the solvent was dried naturally under ambient condition, which was very time consuming and not suitable for large area film fabrication. Here, a rapid GO film formation approach was tested by using the steady hot airflow for the drying of the solvent. We believe this strategy is compatible with roll-to-roll technology. The preparation procedure of the composite electrode is shown in Figure 2a. To illuminate the difference from two approaches for the formation of composite electrode, a relatively high concentration of GO solution (0.5 mg/mL) was used. Figure 2b shows the photo of Ag NW/LGO composite electrodes that were prepared by the two methods with the large area of 6.25 cm^2^. Sample 1 was prepared by a conventional method, in which water was evaporated naturally. Sample 2 was prepared with the assistance of a steady hot airflow, as indicated in Figure 2a. The preparation of Sample 1 was time consuming and resulted in non-uniform film formation, as indicated in Figure 2b. However, with the assistance of steady hot airflow, a flat and uniform GO film could be rapidly fabricated, and the preparation process of the Ag NW/LGO composite was efficient and suitable for large-scale fabrication.

**Figure 3:**
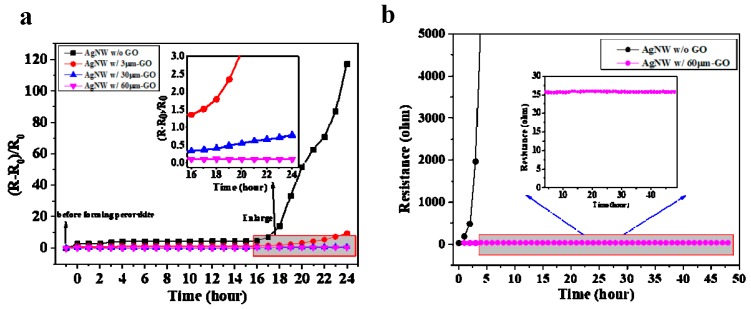
(**a**) The resistance variation of the Ag NW electrodes protected by different sizes of GO sheets (40 μL, 0.25 mg/mL) before and after the fabrication of perovskite devices: and (**b**) the resistance of the Ag NW electrode protected by 60 μm GO sheets under continuous 0.8 V bias and light irradiation (0.5 sun).

To evaluate the anticorrosive effect of the film made of different size of GO sheets, the same volumes of GO aqueous solution with a constant concentration of 0.25 mg/mL were dropped on three identical Ag NW electrodes and allowed to dry under a steady hot airflow, as discussed above. Two electrical contacts using silver paste were formed on the two sides of the electrode to measure its resistance variation before and after device fabrication. PEDOT:PSS layer, perovskite (MAPbI_3_) layer and PCBM layer were fabricated on the composite electrodes in sequence. The control sample of Ag NW electrode without GO was also prepared following the same procedure. The resistance variations of the Ag NW/GO composite electrodes were measured in a glove-box filled with N_2_ (O_2_ <0.1 ppm, H_2_O <0.1 ppm) after device fabrication. As illustrated in Figure 3a, the resistances of all composite electrodes remained stable for the first 10 h, including the control sample. This was because of the glove-box, in which water and oxygen content were low. The process of decomposition of perovskite layer was relatively slow. Meanwhile, the corrosion of halide species against silver nanowires was also a gradual process in such condition. Over time, the resistance of the control electrode changed dramatically, by exhibiting more than 100 times the original resistance during 8 h. This result directly reflected the severe corrosion of the silver nano-network underneath the perovskite film. In contrast, the resistances of the electrodes protected by GO sheets increased slowly, and the resistance increasing rates decreased as the sizes of GO sheets increased. The inset in Figure 3a clearly indicates that the Ag NW electrode protected by 60 μm GO sheets showed an excellent chemical stability, which can keep stable with negligible variation for 24 h. In addition, we also measured the resistance of the Ag NW electrode protected by 60 μm GO sheets under continuous 0.8 V bias and light irradiation (0.5 sun), as shown in Figure 3b. The result clearly demonstrates that, even under continuous electricity and irradiation, the composite electrode was almost unaffected and maintained a stable resistance, proving the outstanding protective effect of large size GO sheets.

**Figure 4:**
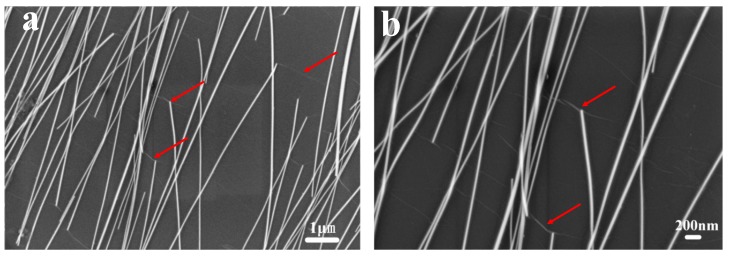
(**a**) Large-scale; and (**b**) enlarged SEM images of silver nano-network under the protection of 60 μm GO sheets. The red arrows inside indicate the crinkles of GO.

**Figure 5:**
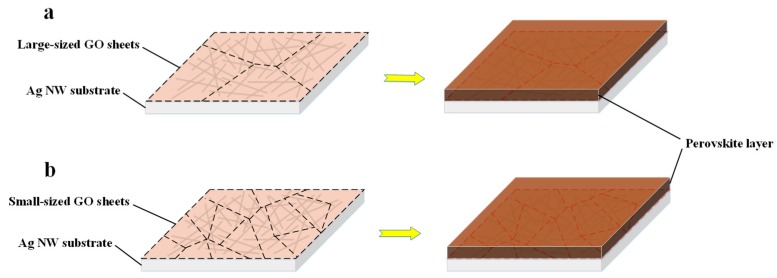
Schematic of Ag NW electrodes protected by: (**a**) large GO sheets; and (**b**) small GO sheets.

To illuminate the enhanced stability of the composite electrode of Ag NW/LGO against perovskite layer, the electrode was characterized by SEM. As evidenced by the SEM images from the silver nano-network coated with 60 μm GO sheets (Figure 4), the silver nanowires were widely covered by single graphene oxide sheet, which acted as anti-corrosive barrier against halide ions from perovskite. Since the average length of the silver nanowires was about 30 μm, it is easy to understand that a larger area of GO sheet can protect more silver nanowires from corrosion. In other words, less GO would be necessary to fully cover the same area of Ag NW electrode than one composed of small-sized GO sheets, as schematically depicted in Figure 5. Due to the physical stacking of the GO sheets, it is also reasonable to believe that the penetration of halide species through the cracks indicated by the dotted lines formed between GO sheets could still happen and caused the deterioration of the electrode. Therefore, the gradual increase of the resistance of electrode composited by 30 μm and 3 μm GO sheets after the fabrication of perovskite layer could be attributed to the formation of more cracks or defects in the anticorrosive film. Stacking more GO sheets on the electrode to block the cracks with additional GO layers might be an efficient method to prevent such penetration; however, this would be harmful to the optical transparency and electrical conductivity of transparent electrode. Solar cells fabricated on the composite electrode based on that strategy would be difficult to optimize to achieve good performance. It should also be noted that, even though the combination of metal nanowire electrode and single- or double-layer graphene in CVD method being able to obtain the best optical, electrical and stability properties, the hydrophobic feature of the film would add difficulty to the fabrication of the charge transporting layer and perovskite film. Therefore, relatively large GO sheets with average size of 60 μm were used for demonstration.

**Figure 6:**
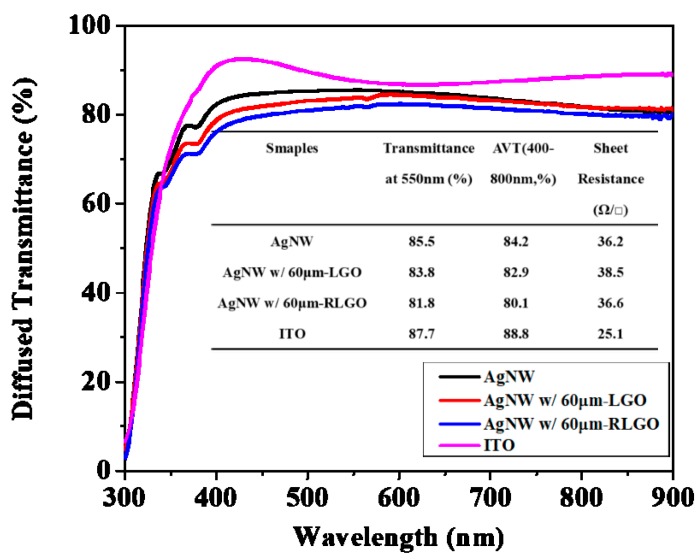
Diffused transmission spectra of silver nano-network electrodes and the network electrode coated by the same amount of 60 μm LGO sheets with and without chemical reduction, and the commercial ITO substrate. The inset is the performance parameters of these samples.

Even though the reduced stacking layer from large size GO sheets helped decrease the vertical resistance between bottom Ag NW electrode and charge transporting layer fabricated above, the lateral resistance of GO was poor due to the high degree of oxidation. Partially-reduced LGO was still essential to improve the lateral conductivity, which could help the efficient collection of photo-induced carriers generated at the position away from the metal nanowires. Hence, an appropriate amount of NaBH_4_ was added into the pristine LGO solution as a reducing agent to enhance the electrical conductivity of LGO film. More details are shown in Experimental Section 2.3. To characterize the optical property of the composite transparent electrodes, diffused transmission spectra of the silver nano-network electrodes coated with 40 μL of LGO (average size of 60 μm) solution with and without partial reduction by optimal amounts of NaBH_4_ are shown in Figure 6. The spectra indicated the composite electrodes of Ag NW/LGO and Ag NW/RLGO including the glass substrate exhibited transmittances of 83.8% and 81.8%, respectively, at the wavelength of 550 nm. Contributed by the size effect of GO sheet, as discussed above, the quantity of GO sheets forming the anticorrosive film on the electrode surface could be reduced. The optical loss of bare silver nanowire electrode brought by the addition of 60 μm LGO and 60 μm RLGO were minimized to 1.6% and 4.6% of average transmittance (AVT), respectively, at the spectral range of 400–800 nm.

**Figure 7:**
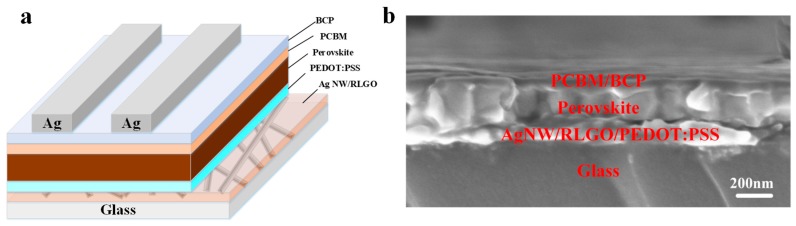
(**a**) Schematic illustration of the perovskite solar cell structure on an Ag NW/LGO composite electrode; and (**b**) cross-sectional SEM image of the perovskite solar cell fabricated on Ag NW/LGO composite electrode.

Based on the excellent chemical stability and transmittance of the Ag NW/LGO composite electrodes, we further evaluated the transparent electrodes for perovskite solar cell applications. To fabricate PVSCs based on the Ag NW nano-network transparent electrodes protected by anti-corrosive LGO film, PEDOT:PSS, MAPBI_3_, PCBM and BCP were deposited as hole transport layer (HTL), absorber layer, electron transport layer (ETL) and buffer layer, respectively. The devices were finally finished after thermal evaporation of the silver counter electrode. The schematic diagram and cross section SEM image are shown in Figure 7a,b. The control devices of perovskite solar cells on commercial ITO electrode were also fabricated for reference. More details on device fabrication can be found in Experiment Section 2.4.

**Figure 8:**
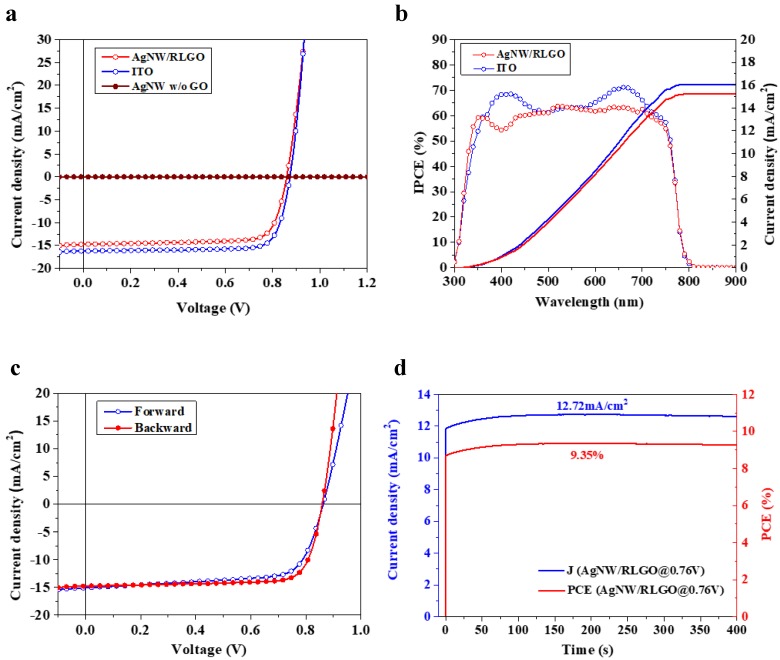
(**a**) J–V curves of the PVSC fabricated on the Ag NW nano-network electrode protected by 60 μm LGO sheets. Data from commercial ITO glass and Ag NW electrode without GO are also presented. (**b**) IPCE performances of the devices fabricated on the nano-composite electrode and ITO substrate. The blue and red solid lines indicate the integrated current of the ITO-based and composite electrode based devices, respectively. (**c**) J–V curves of the PVSC fabricated on the Ag NW nano-network electrode protected by 60 μm LGO sheets measured under forward and backward bias scanning. (**d**) Steady photocurrent (blue) and PCE (red) under 1 sun illumination of the Ag NW/RLGO based PVSC.

The current density–voltage (J–V) curves of PVSCs fabricated on ITO glass, the pristine Ag NW electrode without GO and the composite electrode of Ag NW nano-network with 60 μm LGO sheets are shown in Figure 8a, and the performance parameters are summarized in Table 1. The performance of PVSCs was obtained under 1 sun illumination (AM 1.5G, 100 mW/cm^2^) irradiated from the bottom electrode side covered with a black shadow mask. The perovskite solar cell using the Ag NW/RLGO electrode showed an open circuit voltage (V_OC_) of 0.87 V, short-circuit current (J_SC_) of 15.43 mA/cm^2^, fill factor (FF) of 70.9%, and PCE of 9.62%. The incident photon-to-electron conversion efficiency (IPCE) of the device based on the composite electrode is shown in Figure 8b. The calculated J_SC_ from the IPCE spectrum is 15.23 mA/cm^2^, which is consistent with the value of 15.43 mA/cm^2^ from the J–V curve. Figure 8c exhibits the J–V hysteresis characteristics of the cells based on the optimized Ag NW/RLGO composite electrode using a dwell time of 10 ms for both forward (J_SC_ to V_OC_) and backward (V_OC_ to J_SC_) scan directions. The detailed hysteresis performance parameters are summarized in Table 2. As shown in Table 2, a PCE of 9.62% was achieved in the backward scan, whereas the PCE value was 9.08% in the forward scan. The little hysteresis phenomenon might be due to the coating of the PCBM layer [43,44]. The stabilized photocurrent and PCE at the maximum power output point (MPP) under AM1.5 sun illumination are shown in Figure 8d, which exhibited stabilized photocurrent of 12.72 mA/cm^2^ and PCE of 9.35% at 0.76 V under 400 s continuing light irradiation, agreeing well with the value measured from J–V curves (Figure 8c). As illustrated in Table 1, the J_SC_ value from Ag NW/RLGO composite electrode accounted for 95% of the value of reference device, which was consistent with the optical transparency ratio of the composite electrode and ITO glass, indicating their qualification of optical transparency and electrical conductivity for transparent bottom electrode. The slight decrease of the FF value from the Ag NW/RLGO composite electrode-based device could be attributed to relative larger surface roughness of the composite electrode. Even though the coating of GO and PEDOT:PSS layers could improve the smoothness of bare silver nanowire electrode, the surface of composite electrode in large scale was still worse than the ITO film possessing nanometer surface roughness. The additional GO layer and thick PEDOT:PSS layer slightly affected the charge transferring between the composite electrode and perovskite layer, as evidenced by the slight increase of the serial resistance in Table 1. Nevertheless, a PCE of 9.62% was achieved, which corresponded to nearly 85% of that of the reference device with an ITO electrode, indicating that it is suitable to use our Ag NW/RLGO based electrode for PVSCs. More importantly, our Ag NW composite electrode protected by large size GO sheets exhibited superior chemical stability over those that are pristine or protected by small size GO sheets, and maintained 95.1% average transmittance of pristine Ag NW electrode. Furthermore, the fast GO film formation method, which was assisted by steady hot airflow, was beneficial to large scale fabrication technology. Future optimization on the surface of composite electrode and perovskite film quality on the composite electrode would undoubtedly boost the efficiency of the devices.

## 4. Conclusions

In summary, different sizes of GO sheets from a centrifugal classification method were used and compared for the formation of fully-covered barrier on silver nanowire electrode with the assistance of steady hot airflow. Experimental results show that the size of GO sheet played an important role in preventing the corrosion of halogen species. By employing large size GO sheets, the anti-corrosive ability of Ag NW/LGO composite electrodes were significantly improved compared to those with small size GO sheets, as proven by monitoring the resistance variation under external bias and light illumination. Moreover, the Ag NW/RLGO composite electrode exhibited excellent transmittance of 81.8% at 550 nm and maintained 95.1% average transmittance (AVT) of the pristine Ag NW electrode. Consequently, the PVSCs fabricated on the Ag NW/RLGO based composite electrodes achieved a power conversion efficiency (PCE) of 9.62%, i.e., nearly 85% of the PCE from the solar cell on commercial ITO. These results clearly demonstrate the potential of Ag NW/RLGO composite as window electrode for PVSCs, which would be beneficial to large-scale low-temperature fabrication and future commercialization of PVSCs.

## Figures and Tables

**Table 1:** Summarized photovoltaic parameters of PVSC devices based on Ag NW/RLGO composite electrodes, control ITO substrates and pristine Ag NW electrodes without GO.

Samples	V_oc_ (V)	J_sc_ (mA/cm^2^)	FF (%)	PCE (%)	Rs (Ω cm^2^)
Ag NW/RLGO	0.87	15.43	70.9	9.62	98.85
Average	0.86 ± 0.01	14.82 ± 0.59	69.7 ± 4.2	8.89 ± 0.67	96.79 ± 24.61
ITO	0.87	16.23	80	11.34	54.49
Average	0.87 ± 0.02	15.98 ± 0.62	78.1 ± 1.5	10.87 ± 0.35	61.54 ± 11.42
Ag NW without GO	0.04185	0.000026	30.66	5.12 × 10^−9^	1.38 × 10^8^
Average	0.037 ± 0.009	(21.2 ± 5.4) × 10^−6^	27.41 ± 2.1	(3.48 ± 1.27) × 10^−9^	(1.58 ± 0.19) × 10^8^

**Table 2:** Photovoltaic parameters of PVSC devices based on Ag NW/RLGO composite electrodes scanned forward and scanned backward.

Samples	V_oc_ (V)	J_sc_ (mA/cm^2^)	FF (%)	PCE (%)
Forward	0.86	15.08	69.8	9.08
Backward	0.87	15.43	70.9	9.62
